# Supplementary material for: Back Pain without Disease or Substantial Injury in Children and Adolescents: A Twin Family Study Investigating Genetic Influence and Associations
Source: Children (Basel). 2023 Feb 14;10(2):375. doi: 10.3390/children10020375 (PMC9955700; doi:10.3390/children10020375)
Supplement: Supplementary file 1 [file children-10-00375-s001.zip › children-1974010-supplementary.pdf]

Supplementary Information

Appendix 1: Questionnaires back pain and zygosity

BACK PAIN AND OTHER REGIONAL PAIN DISORDERS IN AUSTRALIAN TWIN FAMILIES

Full name: ____

Please indicate whether you are the mother/father/guardian/twin/sibling: _________

Date of birth (dd/mm/yyyy): ____________________

Sex (male/female): __________________

1. Have you had pain for most of the **last month** in any parts of your body? ☐ Yes ☐ No

If you answered "**Yes**", please mark the parts of your body where you have been experiencing those pains. If more than one body part is affected, please show which has been the **most important** pain area.

If you answered **"No",** please go to Question 3.

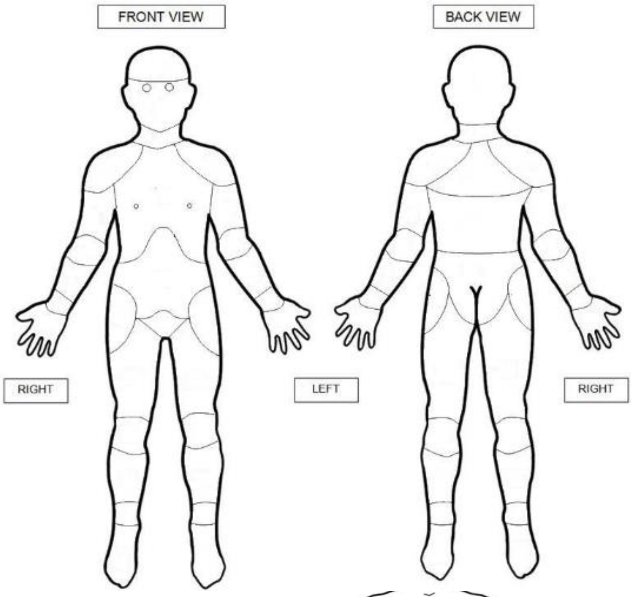

2. Were these pains caused by injury (e.g., a fall or sport injury)? ☐ Yes ☐ No

3. During your lifetime, have you ever had pain in your low back
in the shaded area, which lasted for at least 3 months? ☐ Yes ☐ No

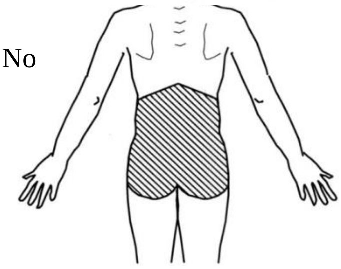

If you answered "No" to Question 3,
you do not need to answer any further questions on this sheet.

4. Was the low back pain caused by injury (e.g., a fall or lifting a heavy weight)? ☐Yes ☐ No

5. Has low back pain occurred from time to time during your life without obvious reason? ☐Yes ☐ No

6. How old were you when you first had trouble with your back? ____ years

7. Does/did your low-back pain ever spread down your leg? ☐ Yes☐ No

8. Has your low-back pain ever stopped you from going to school or playing sports? ☐ Yes☐ No

9. In the last 6 months, how often have you had low-back pain? (tick one)

☐	☐	☐	☐	☐
About every day	More than once a week	About every week	About every month	Rarely or never

ZYGOSITY QUESTIONNAIRE FOR TWINS

Question 1: Are your twins the same sex?

☐ Yes - Please go to **Question 2**

☐ No - Thank you, **that completes our zygosity survey**, please complete the remaining questionnaires with the ATR Response form and consent in the reply paid envelope provided

Question 2: Have your twins been DNA tested to confirm whether they are identical (monozygous) or non-identical (dizygous)?

☐ Yes - The DNA test showed that the twins are: ☐ Identical ☐ Non-identical
Thank you, **that completes our survey**, please return this with the ATR Response form in the reply paid envelope provided

☐ No - They have not had a DNA test – please go to **Question 3**

Question 3: Please tick the appropriate box for each of the following questions;

Are your twins mistaken for each other by people who know them?

☐ Frequently
☐ Sometimes
☐ Rarely
☐ Never

a) Non-identical twins are no more alike than ordinary brothers or sisters. On the other hand, identical twins have such strong physical likeness to each other in height, colouring, facial features, etc., that people often mistake one for the other or say they are "as alike as two peas in a pod". Having heard these statements, do you think your twins are physically identical twins or non-identical twins?

☐ Physically identical
☐ Non-identical

b) Based on your last answer, please rank the following items in the order that they influenced your answer. The most influential factor should be ranked number 1, the next number 2, with the least influential factor as number 3.

_____ I just know.
_____ What the physician told me about the placenta.
_____ The overall similarity or dissimilarity of their physical features.

c) Do you think your twins are identical or fraternal?	☐ Identical	☐ Fraternal
d) Are you sure that your twins are (above answer)?	☐ Yes	☐ No
e) Are the children as alike as two peas in a pod?	☐ Yes	☐ No
f) Are the children of only ordinary family resemblance?	☐ Yes	☐ No
g) Who can identify each twin		
- parents?	☐ Yes	☐ No
- brothers and sisters?	☐ Yes	☐ No
- teachers?	☐ Yes	☐ No
- friends?	☐ Yes	☐ No
- strangers?	☐ Yes	☐ No
h) Are there differences in your twins' hair colours?	☐ Yes	☐ No
i) Are there differences in your twins' eye colours?	☐ Yes	☐ No

Thank you, that completes this questionnaire.

Appendix 2: Criteria for common pain disorders of childhood without disease

<u>Classification of growing pains (GP)</u> (35)

Criteria for the diagnosis of GP

Essential criteria

1. Pain in both legs
2. Pain began between the ages of 3-12 years old
3. Pain typically occurred at the end of the day or during the night
4. There was no significant limitation of activity and no limping

Excluding factors

1. A pattern of pain severity not consistent with GP
2. Any indication of a definite orthopaedic disorder
3. Any abnormalities on specific testing (eg X-rays, bone scans, blood tests)

Additional descriptive features of growing pains

1. Pain persisted at least three months
2. There were periods of days, weeks or months without leg pains
3. Pain not a problem in the morning
4. There was no associated lack of well-being

Definite GP: at least fulfilling 3 essential criteria, and absent excluding factors

<u>Classification of restless legs syndrome (RLS)</u> (39)

Criteria for the diagnosis of RLS

Essential criteria

1. The urge to move the legs, which may be accompanied by unpleasant or uncomfortable sensations
2. The urge to move begins or worsens with sitting or lying down
3. The urge to move is partially or totally relieved by movement
4. The urge to move is worse in the evening or night than during the day, or occurs exclusively in the evening or night

AND: the child uses his or her own words to describe leg discomfort

Supportive criteria

1. Sleep disturbance is inappropriate for age
2. A biological parent or sibling has definite RLS
3. A sleep study has documented a Periodic Leg Movement index of $\geq$ 5/hours of sleep

Definite RLS: all essential criteria or 1-4 essential criteria and 2-3 supportive criteria

<u>Classification of (migrainous) headache, based on IHS Classification ICHD-II</u> (33)

Paediatric criteria (used for classification in twins and siblings)

Migraine with aura:

A. At least 5 attacks fulfilling criteria B-D
B. Headache attacks lasting 1-72 hours
C. At least 2 of the following headache characteristics:
    a. Bifrontal/bitemporal or unilateral location
    b. Pulsation/throbbing quality
    c. Moderate or severe pain intensity
    d. Aggravation by or causing avoidance of routine by physical activity
D. During headache, at least 1 of the following (may be inferred from their behaviour)
    a. Nausea and/or vomiting
    b. Photophobia and phonophobia
E. Visual and/or sensory and/or speech symptoms occurring before headache
F. Not attributed to another disorder

Adult criteria (used for classification in parents)

Migraine with aura:

A. At least 5 attacks fulfilling criteria B-D
B. Headache attacks lasting 4-72 hours
C. At least 2 of the following headache characteristics:
    a. Unilateral location
    b. Pulsation/throbbing quality
    c. Moderate or severe pain intensity
    d. Aggravation by or causing avoidance of routine by physical activity
D. During headache, at least 1 of the following
    a. Nausea and/or vomiting
    b. Photophobia and phonophobia
E. Visual and/or sensory and/or speech symptoms occurring before headache
F. Not attributed to another disorder

Migraine without aura:	criteria as above, except for criterion E
Non-migraine headache:	recurrent headache attacks lasting 1-72 hours (children) or 4-72 hours (adults), but not fulfilling all criteria of migraine
